# Inhalation of Oxygen for Therapeutical Purposes

**Published:** 1893-12-02

**Authors:** Benjamin Ward Richardson


### Inhalation of Oxygen for Therapeutical Purposes.

By Sir Benjamin Ward Richardson, M.D., F.R.S.

I have recently been using, in hospital, oxygen gas as a remedy administered by inhalation. It is the revival of an old study of mine which I should like to leave in a more satisfactory state than I found it and than it is in at this moment. I do not think anyone can as yet claim that he has proved a definite and distinct place for oxygen in the treatment of disease. When we administer a gas like nitrous oxide or a vapour like chloroform we know what we are about. We feel sure when we administer that we shall produce torpor or sleep, and we can divide the symptoms we produce them into degrees or stages, feeling that every other practitioner can do the same. This is rational, we really may say mathematical practice, and if we could bring all processes of a therapeutical kind to the same certainty we should establish a positive science. We might be left to differ on particulars; we might differ in respect to the merits or safety of particular agents; but these would be little more than details we should quickly master with a steadfast knowledge of our art in all great principles. I do not doubt that the day will come when we shall universally treat disease by medical inhalation. It is the readiest method. The grand portal or entry into the blood currents, and so into the system at large, is by the lesser circulation.

The lungs are a direct part of the circulation, and to make them the gates of remedies is as philosophical as it is practical. "Respiration is the second digestion," said Arbuthnott, and in the second digestion we have a means for introducing medicinal substances without disturbing the first, a great advancement.

When oxygen was discovered by Priestly as a constituent of the atmosphere, and as the supporter of life or the fire of life, men called out Eureka, as if the very elixir vitæ had been found. In a short time the breathing of oxygen became a popular remedy, and pneumatic medicine, like pneumatic chemistry, had its birth. There is quite a lost literature on the treatment of disease by the inhalation of oxygen. Hill, one of the early experimenters on this subject, was a remarkable enthusiast, and, as I think, was really a sound and careful explorer. He employed the gas most in scrofulous and tuberculous patients, and as his results were honestly reported, there need be no wonder that he was satisfied, some of his labours being distinctly successful. It is strange that he did not establish a school. He did not; and if we except Beddoes, whose genius met with a short-lived reputation, no one did much in a practical direction. In time it began to be suspected that oxygen was merely a temporary remedy, too troublesome to make as to be always ready, and too uncertain to be relied upon. In brief, there never was such a demand for it as to bring it into commerce. The travellers of the great drug houses carried specimens of all medical appliances that were marketable, but oxygen did not find its way into their lists, and so it was forgotten. It is only within the last few years, only since Faraday led the way towards the art of compressing gases, and making them transportable under pressure, that we have had the promising remedy at command.

It must be admitted, too, that there was some considerable reason for failure of faith in oxygen as a remedy, even when it was administered with true skill and perseverance. The idea about it was that it seemed to do more than it did. Chaptal, speaking of it in the treatment of phthisis pulmonalis, said with almost poetic beauty, "It strewed flowers to the grave," a remark that carried, and conveyed, in telling form, a common experience.

Where there is uncertainty there is never any progress, and to this day our grand incompetency in therapeutics rests on uncertainty. The rubbish of remedies that is carted to our medical dust-bins day by day has its source in uncertainty. We, probably, should not require two dozen remedies altogether to meet every form and phase of disease if we were as sure of doing what we want to do with each as we are sure with nitrous oxide or chloroform. We are uncertain about oxygen, and oxygen therefore lies still aside a doubtful curative substance, speculative, unsound. The difficulty about oxygen and its uncertainty is an exposition of our own ignorance as to the nature of it and the use of it. Let me give an illustration. I selected two cases of early phthisis. The patients were girls of the same age, and in general characteristics were of the same construction. They were in the same stage of the disease. They presented the same temperature. They were in the same position; they occupied bed by bed; they were subjected on the same day to the same treatment—inhalation of oxygen. The supply of oxygen was from the same source; the mode and times of inhalation were the same, and no other remedy was allowed to interfere. They were, in short, treated exclusively by the inhalation of oxygen under every condition of diet and regimen alike. They were, moreover, willing subjects; they did precisely what they were told. The test was as crucial as, I believe, it could be, but there was no correspondence as to the results. In one instance under the treatment the patient began to recover in the most satisfactory manner. The hacking cough ceased; the crepitation in the left lung, which was the seat of the mischief, gradually ceased. The digestion which had been imperfect improved; food was taken in larger quantities, and with desire as well as relish; weight gained; strength increased; and, in the end, after a period of eight weeks, a most striking recovery was presented. In the other patient no such favourable changes took place. For the first few days there seemed an improvement, then all went again to the bad; the cough increased, the lung mischief gradually spread, the digestion failed, and the emaciation was more pronounced. Suddenly an attack of pericarditis complicated the condition, and although to the administration of oxygen other measures were employed, it was useless in arresting the fatal course of the disease. No experiences could be more definite than these—none more contradictory. In one instance oxygen appeared to cure; in the other its action was, at best, entirely negative. Is it wonderful that different men, seeing such differences as these, should declare oxygen a divine remedy, a specific; should declare oxygen an absurd remedy, an impostor? Candidly, both cases only prove our own ignorance. Why they do so I will try to explain, respectfully, in another chapter.

